# Retraction: MiR-34b inhibits the proliferation and promotes apoptosis in colon cancer cells by targeting Wnt/β-catenin signaling pathway

**DOI:** 10.1042/BSR-2019-1799_RET

**Published:** 2024-04-29

**Keywords:** Colon cancer, Invasion, Migration, MiR-34b, Proliferation

This article is being retracted from *Bioscience Reports* at the request of the authors following receipt of a notification from a reader, alerting the Editorial Office to images in Figures 3A and 3B that have also been published in Zhuang et al 2019 (doi: 10.26355/eurrev_201903_17244). Further analysis of the images in Figure 3B in the Bioscience Reports article has revealed duplicated regions within the images.

The authors wish to retract the article. The Editor-in-Chief and Editorial Board agree with the retraction.

